# Electromagnetic Field Enhancement of Nanostructured TiN Electrodes Probed with Surface-Enhanced Raman Spectroscopy

**DOI:** 10.3390/s22020487

**Published:** 2022-01-09

**Authors:** Ibrahim Halil Öner, Christin David, Christine Joy Querebillo, Inez M. Weidinger, Khoa Hoang Ly

**Affiliations:** 1 Fakultät für Chemie und Lebensmittelchemie, Technische Universität Dresden, Andreas-Schubert-Bau, Zellescher Weg 19, 01069 Dresden, Germany; ibrahim.halil.oener@me.com (I.H.Ö.); christine_joy.querebillo@tu-dresden.de (C.J.Q.); 2 Abbe Center of Photonics, Institute of Condensed Matter Theory and Optics, Friedrich-Schiller-Universität Jena, Max-Wien-Platz 1, 07743 Jena, Germany; christin.david@uni-jena.de

**Keywords:** titanium nitride, nanotubes, plasmonics, electromagnetic field enhancement, surface enhanced Raman scattering spectroscopy

## Abstract

We present a facile approach for the determination of the electromagnetic field enhancement of nanostructured TiN electrodes. As model system, TiN with partially collapsed nanotube structure obtained from nitridation of TiO_2_ nanotube arrays was used. Using surface-enhanced Raman scattering (SERS) spectroscopy, the electromagnetic field enhancement factors (EFs) of the substrate across the optical region were determined. The non-surface binding SERS reporter group azidobenzene was chosen, for which contributions from the chemical enhancement effect can be minimized. Derived EFs correlated with the electronic absorption profile and reached 3.9 at 786 nm excitation. Near-field enhancement and far-field absorption simulated with rigorous coupled wave analysis showed good agreement with the experimental observations. The major optical activity of TiN was concluded to originate from collective localized plasmonic modes at ca. 700 nm arising from the specific nanostructure.

## 1. Introduction

Titanium nitride (TiN) is an emerging substrate material for optical and sensing applications [1,2]. This low-cost plasmonic active transition metal nitride exhibits several relevant material properties, such as metal-like conductivity, high thermal and chemical stability as well as high biocompatibility, rendering TiN a unique refractory plasmonic material and potential alternative to traditionally employed plasmonic substrates, such as silver and gold [1,3,4]. So far, TiN has been featured as a corrosion-stable electrode in bioelectronic/biomedical devices [3,4], as a support material for electrocatalysis [5] and photocatalysis [6], as a refractory metamaterial [7], as heat conversion material in thermal devices [8], as well as sensor material for optical spectroscopies [9,10,11].

TiN’s capability to function as an optical sensor sensitively depends on the quality of its plasmonic near-field light enhancement upon illumination. The surface plasmon resonance (LSPR) is typically located in the visible to near-IR region and can be tuned with respect to resonance frequency and magnitude by tailoring the material’s nanostructure [12,13]. In this respect, various TiN nanostructures have been investigated for their light modulation aptitude, including nanoparticle assemblies [14,15], nanocubes [16], nanotubes [17], nanorods [11,13], as well as thin films of TiN [18]. Each system has shown specific plasmonic activity that can be further fine-tuned by controlling structural parameters.

Characterization of the plasmonic activity of TiN nanostructures largely relies on UV-Vis spectroscopy, which monitors the overall optical response of the material. This is often combined with theoretical simulations to rationalize the experimental data [19]. While such information is crucial for understanding the optical properties, UV-Vis spectra do not necessarily provide direct information on the strength of the near field enhancement nor can the light enhancement be directly quantified from the experimental data. Moreover, UV-Vis spectra are recorded in air or vacuum, but typically not under conditions at which a TiN-based sensor would potentially operate, e.g., in electrolyte solutions and/or under an applied electrical potential.

Surface-enhanced Raman scattering (SERS) spectroscopy can deliver this information. This versatile technique is highly sensitive and surface-selective and, thus, has been widely employed [20], e.g., for investigation of surface reactions [21] and as a detection method in various optical sensor devices [22]. SERS spectroscopy exploits amplified electromagnetic fields provided by certain substrates, such as a plasmonic material, for enhancing Raman signals of molecules located within [23]. The relative enhancement of the Raman signal is expressed as Raman enhancement factor (REF) and, in the absence of other signal enhancement mechanisms, is related to the relative increase in field strength, expressed as electromagnetic field enhancement factor (EF), via:

(1) $$\sqrt{REF}=EF$$

By exploiting this relationship, the near-field enhancement of a substrate can be quantified by determining the REF of a suitable probe molecule in a SERS experiment. However, other relevant factors can also affect the Raman signal. First and foremost, the chemical enhancement effect can afford dramatic changes in the Raman spectrum [24]. This effect is based on chemical interactions between the probe molecule and the surface that leads to altered molecule properties and, hence, Raman scattering efficiencies.

TiN’s intrinsic plasmonic near-field enhancement has sparked its application as a SERS substrate [25]. REFs ranging up to 10^4^ have been reported for certain probe molecules loaded on TiN nanostructures [1,13,26,27]. However, in most cases the experimental parameters do not allow for a direct correlation between REF and EF, as stated by Equation (1) and thus, for drawing conclusions on the magnitude of the electromagnetic field enhancement (EME) of the respective support. Specifically, the probe molecules have been directly adsorbed/chemosorbed as dried films onto the TiN metallic surface. Moreover, strongly absorbing dyes, such as rhodamine 6G or methylene blue [28] have been used as probe molecules and resonantly excited, exploiting additional Raman signal enhancement via the resonance Raman effect. As such, a large contribution from the chemical enhancement effect is expected, such that the observed Raman signal amplification does not strictly correlate with the EME at the TiN surfaces anymore.

We demonstrate a facile experimental procedure based on SERS spectroscopy to exclusively assess the light amplification of nanostructured TiN electrodes. As a model system, we introduce TiN with a partially collapsed nanotubular structure. This system exhibits a highly complex nanostructuring with local periodicity and, as such, allows to visualize plasmonic and light trapping contributions to the near-field enhancement. We show that by using a non-surface binding/interacting SERS-reporter molecule, effects of chemical enhancement can be minimized. In conjunction with electromagnetic field calculations based on rigorous coupled wave analysis, we describe a coherent approach for probing the near-field enhancement and to derive information on the nature of the light modulation by TiN.

## 2. Materials and Methods

Azidobenzene (AB) (purity ≥ 95%) and tert-butylmethylether (tBME) (purity 99.8%) were purchased from Sigma Aldrich and used without further purification. Nanostructured TiN electrodes were obtained by ammonolysis of TiO_2_ nanotubes (TiO_2_ NTs) at 950 °C for 3 h at a heating rate of 300 K·h^−1^ and ammonia flow rate of 10 L h^−1^. The preparation of the TiO_2_ NTs followed described protocols [29]. Briefly, Ti foils were anodized for 2 h at 20 V in 0.202 M ammonium fluoride in glycerol:H_2_O 50:50 vol. at room temperature, followed by calcination in air at 450 °C.

### 2.1. Surface Enhanced Raman Scattering Measurements

Raman and surface-enhanced Raman scattering measurements were carried out using a LabRamII (Horiba, Potsdam, Germany) spectrometer equipped with a nitrogen-cooled Symphony CCD detector (Horiba, Potsdam, Germany) as well as a S&I Monovista spectrometer (S&I GmbH, Dresden, Germany) equipped with a nitrogen-cooled Pylon CCD detector (Teledyne Princeton Instruments, Trenton, United States). The laser light was focused on the sample using a 20× objective with a numerical aperture of 0.35 (Nikon, Amsterdam, The Netherlands). The laser power was set to 1.0 mW. Accumulation time was 10 s. After each measurement, the TiN electrode was rinsed with tBME and Millipore water and dried under N_2_ stream. All experiments were carried out at −20 °C using a THMS600 cryostage (Linkam, New Town, UK). Excitation wavelengths were chosen at 413 nm (Innova, Coherent Inc., Santa Clara, CA, USA), 514 nm (Innova 300c, Coherent Inc., Santa Clara, CA, USA), 594 nm (MamboTM 100, Hübner Photonics GmbH, Kassel, Germany), 647 nm (Innova 300c, Coherent Inc., Santa Clara, CA, USA), and 785 nm (Hübner Photonics GmbH, Kassel, Germany) laser excitation. UV-Vis spectra were acquired using a CARY 4000 spectrometer (Agilent Technologies, Santa Clara, CA, USA) equipped with a reflection unit.

### 2.2. Calculations

Optical properties of the TiN nanostructures were investigated in silico using rigorous coupled wave analysis (RCWA). As for model nano-geometry, regular arrays of upright, thin-walled, hollow TiN nanotubes were used [26,29]. The optical response in terms of near-field enhancement ${\left|\vec{E}\right|}^{4}/{\left|{\vec{E}}_{0}\right|}^{4}$ and far-field absorbance A=1−R−T relative to the response of the flat TiN substrate were calculated for several nanotube lengths d. $\vec{E}$ and ${\vec{E}}_{0}$ represent the local electromagnetic field vector upon excitation and the field vector of the incident laser beam, respectively. R and T denote reflectivity and transmission, respectively. The optical parameters of TiN were taken from [12]. Near-field electromagnetic enhancement ${\left|\vec{E}\right|}^{4}/{\left|{\vec{E}}_{0}\right|}^{4}$ were calculated by averaging the local fields over a unit cell of each regular structure individually. The change in dielectric constant (air in the UV-Vis spectra) to n=1.3364  (tMBE in the Raman measurements) was considered. Far-field simulations (reflection and transmission) were conducted with the structure in air.

## 3. Results

### 3.1. Synthesis

After nitridation, the electrodes exhibited a metallic shining typical for TiN (Figure S1A). Raman spectra of the electrodes featured the first-order transversal and longitudinal acoustic modes at 197 and 317 cm^−1^, the first-order optical mode at 555 cm^−1^ as well as the second-order longitudinal acoustic mode at 598 cm^−1^ (Figure S1B) of TiN, confirming the successful transformation from TiO_2_ to TiN [27]. The SEM image of the TiN electrodes revealed a rough nanostructure with collapsed and partially preserved ordered nanotubes of the initial TiO_2_ template (Figure S2).

### 3.2. Optical Characterization

**Figure 1:**
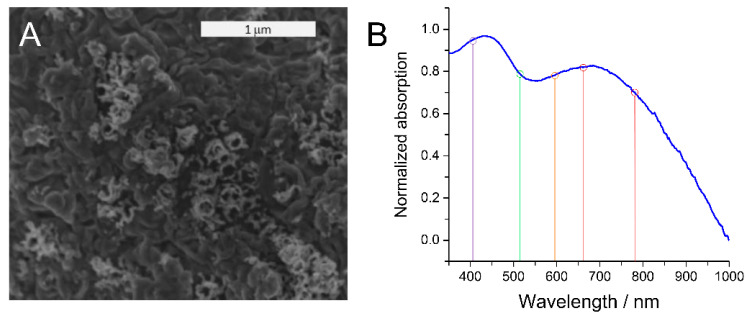
(**A**) SEM image of the TiN electrode created from TiO_2_ nanotubes. (**B**) UV-Vis spectrum of the nanostructured TiN electrode. Colored vertical lines indicate the laser excitation wavelengths in the SERS experiments for determination of the corresponding electromagnetic near-field enhancement.

Optical characterization was carried out using UV-Vis spectroscopy in reflection mode. Two broad absorption peaks centered at ca. 430 nm and ca. 700 nm were observed in the absorption spectrum (Figure 1B), indicating optical activity over a large fraction of the visible spectrum. Previous studies have reported similar absorption profiles for nanostructured TiN and attributed those to the distinct plasmonic activity of the material [1,15,28]. LSPRs of TiN created upon light illumination have been proposed to manifest as broad absorption features that are located at specific frequencies characteristic for the specific nanostructure geometry. This shows that the created nanostructured TiN electrode exhibits high plasmonic activity that is comparable to previously presented systems in the literature.

### 3.3. Electromagnetic Field Enhancement Determination

**Figure 2:**
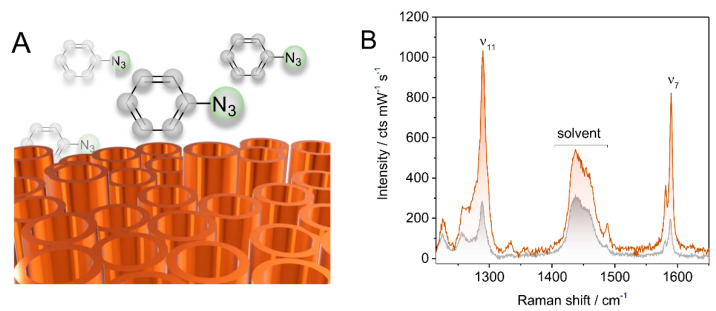
(**A**) Schematic representation of azidobenzene on TiN. (**B**) 647 nm Raman spectra of 0.5 M azidobenzene in tBME/acetonitrile in solution (grey trace) and at the TiN surface (orange trace), respectively.

Next, the near-field enhancement arising from its plasmonic activity of the TiN electrode was experimentally quantified with SERS spectroscopy by exploiting the Raman signal amplification in the presence of elevated fields. In the absence of other enhancement mechanisms, EME is the sole contributor, allowing for the field enhancement factor (EF) to be derived via the REF of a probe molecule as stated by Equation (1). As the field reporter group, the molecule azidobenzene (AB) was chosen (Figure 2A). This compound was found to undergo non-covalent interactions with TiN as verified by cyclic voltammetry experiments. Cyclic voltammograms (CVs) of the TiN electrode showed a reversible increase of the capacitive current after AB incubation (Figure S3). The initial CV curve could be restored by solely rinsing with water, indicating that chemosorption of AB, i.e., strong and permanent attachment of AB to the TiN surface via formation of chemical bond (s), has not occurred.

The Raman spectrum of AB at varied concentrations in tBME revealed two characteristic vibrational modes located at 1290 cm^−1^ and 1590 cm^−1^ (ν_7_ and ν_11_, respectively) with reasonable intensities to be used as marker bands (Figure S4). Determination of the REF for these bands was achieved by comparing the respective band intensities when the laser was focused into the bulk solution (Raman experiment) and when focused on the TiN electrode surface (SERS experiment). Corresponding spectra obtained for both cases at 647 nm laser excitation are exemplarily shown in Figure 2B.

**Figure 3:**
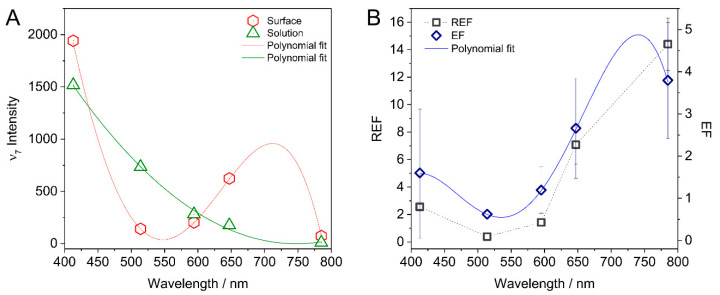
(**A**) Raman intensity of the ν_7_ mode of AB in solution (green) and at the TiN surface (red) as a function of laser excitation wavelength. (**B**) Raman enhancement factor (REF) (blue) and enhancement factor (EF) (black) calculated from the data according to Equation (1) as a function of excitation wavelength. Lines correspond to polynomial fits to the data set.

Mapping the EME of the TiN electrodes over the optical region was achieved by conducting SERS measurements at five representative laser excitation wavelengths between 413 nm to 785 nm (Figure 1B, colored lines). A distinctly different wavelength dependency of the Raman signal intensity in solution and at the TiN surface was obtained. In the bulk solution, the intensity of the Raman spectra was found to increase with lower wavelengths (Figure 3A, shown specifically for the ν_7_ band), as expected for ordinary Raman scattering. A distinctly different trend was obtained when the laser was focused on the TiN electrode. Here, high spectral intensity was observed for 413 nm and 647 nm laser excitation. The Raman scattering efficiency of AB close to the TiN surface was found to correlate with the absorption profile of the TiN electrode, exhibiting a minimum in the green region.

REFs were subsequently derived by comparing the signal intensities of AB obtained from Raman and SERS measurements based on following considerations and according to previously employed procedures: upon assuming that the AB concentration in solution and close to the surface is equal, the number of molecules probed with the laser can be estimated. Here, the Gaussian profile of the laser is approximated by a cylinder with a radius of *r* = 2 mm and a height of ca. *h* = 60 mm [30,31]. In the case where the laser was focused on the electrode surface, only the upper part of the Gaussian beam is considered, leading to a height of ca. 30 mm (Figure S5). Following these assumptions, the wavelength dependent Raman enhancement factor (REF) was calculated by:

(2) $$REF=2\frac{I_{\mathrm{surf}}}{I_{\mathrm{sol}}}$$

$I_{\mathrm{surf}}$ and $I_{\mathrm{sol}}$ denote the Raman intensity at the surface and in the solution. The so-derived REFs are plotted as a function of excitation wavelength in Figure 3B. Also here, the REFs correlate with the UV-Vis absorption profile of the TiN electrode. Specifically, a minimum REF (<1) was found for the 514 nm excitation. Going to higher as well as lower wavelengths afforded an increase of the REF. The highest REF of ca. 14 was measured at 785 nm excitation. The EME expressed as EF were calculated by taking the square root of the REFs (Figure 3B, hollow diamonds) upon assuming the absence of other contributing mechanisms to the Raman signal enhancement [29]. The highest value of ca. 3.9 was obtained at 785 nm, corresponding to an increase in field strength by this factor.

### 3.4. Electromagnetic Field Calculations

**Figure 4:**
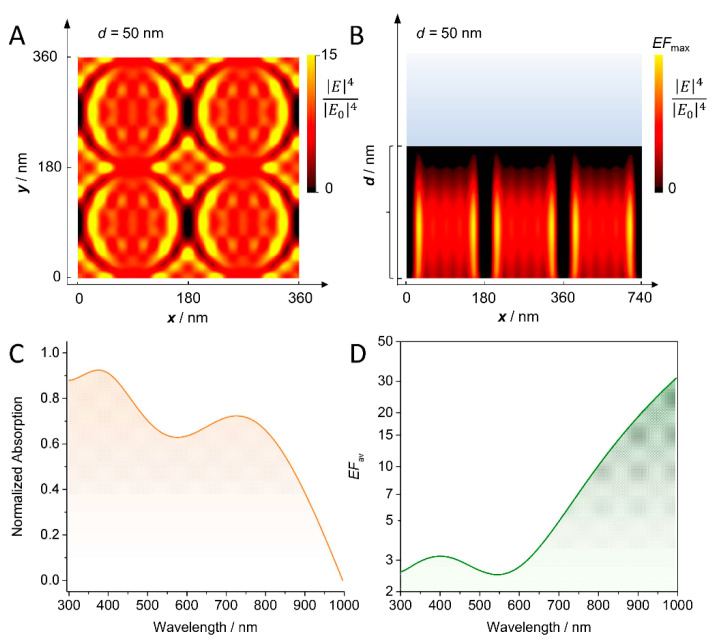
(**A**) Top view over 4 unit cells and (**B**) side view over 3 unit cells showing the distribution of localized field hot spots upon illumination at 413 nm for a nanotube length d=50 nm. The scale shows the field enhancement (EF) value to fourth order $\frac{{\left|\vec{E}\right|}^{4}}{{\left|{\vec{E}}_{0}\right|}^{4}}$. (**C**) Calculated relative absorbance A=1−R−T averaged over all considered nanotube lengths. (**D**) Calculated EFs $\frac{{\left|\vec{E}\right|}^{4}}{{\left|{\vec{E}}_{0}\right|}^{4}}$ averaged over all considered nanotube lengths.

Optical properties of the TiN electrode were simulated using rigorous coupled wave analysis (RCWA). A regular nanotube array with fixed outer tube radius (80 nm) and wall thickness (10 nm) and variable length *d* from 0 (reference planar TiN substrate) to 300 nm was considered as model geometry. These parameters were chosen based on the nanogeometry of the starting TiO_2_ nanotube [29]. For each length, a specific pattern of local field hot spots was obtained (Figure 4A,B, Figures S6 and S7). The highest field enhancement was found at the inner side of the nanotube walls and additionally along the outer walls at the contact points between two nanotubes forming a dipolar pattern along light polarization. For shorter tubes, the hot spots are located along the walls, while for longer tubes, the hot spots are found more progressively closer to the bottom, i.e., at the edge between the TiN nanotube and the Ti substrate. The different patterns originate from interference effects of the light within the nanotube arrays [29].

To compare with experimental data, first the relative absorbance spectra were calculated in far-field simulations for different nanotube lengths. All individual spectra, as well as the averaged spectrum, featured two absorption peaks with maxima at ca. 700 and 400 nm (Figure 4C and Figure S8A), respectively, matching the experimental observations. The position of the respective maxima was found to vary depending on the nanotube length, in line with previous calculations on TiN nanorods in the literature [28]. Figure S9 compares the normalized absorption of the flat TiN substrate to the absorption of an array of hollow TiN nanotubes of 250 nm length. The flat TiN substrate was found to support a surface plasmon polariton (SPP) at a wavelength slightly below 400 nm, which is slightly shifted to about 450 nm in the setup with TiN nanotubes due to the increase in the effective refractive index of the nanostructured region above. Additionally, the TiN electrode showed a strong increase in absorption at ca. 700 nm. This mode arises from the (regular) lattice giving rise to collective surface plasmon resonances [18] that enable the nanostructured TiN support to host additional resonance modes, thereby increasing the light trapping in and around the electrode.

Next, the near-field enhancement of the TiN nanostructures was calculated by averaging the local fields over a unit cell of each regular structure individually. For all individually set tube lengths, a modulation with two ${\left|\vec{E}\right|}^{4}/{\left|{\vec{E}}_{0}\right|}^{4}$ maxima was obtained as observed in the experiment (Figure S8B); albeit none of the performed simulations fully reproduced the position of the observed minima and maxima. The calculated EME factors vary between 2 and 15 and, therefore, lie in the same range as the experimentally derived values. Averaging over several tube lengths yielded an ${\left|\vec{E}\right|}^{4}/{\left|{\vec{E}}_{0}\right|}^{4}$ distribution that shows the same trend as the experimentally derived REF values with a minimum in surface enhancement at around 550 nm (Figure 4D).

## 4. Discussion

We have shown that nanostructured TiN electrodes created from TiO_2_ nanotube arrays as precursors are capable of light enhancement upon illumination. To assess the material’s EME, the REF of the reporter molecule AB was determined with SERS spectroscopy. The wavelength-dependent EF of the TiN electrode could be obtained by taking the square root of the respective REF according to Equation (1), assuming that the electromagnetic enhancement mechanism is the major contributor to the Raman signal enhancement. This assumption is justified considering the gathered experimental results. First, the molecule AB is proposed to accumulate at the TiN surface without undergoing chemisorption. Instead, physisorption was concluded from CV experiments that showed the removal of AB from the TiN surface by solely rinsing with water (Figure S3). Second, no differences between the Raman spectrum of AB in solution and its SERS spectrum were noted (Figure 2B), confirming a conserved molecular integrity at/near the TiN interface and, hence, excluding a direct binding to TiN. Due to the absence of chemical interactions, enhancement of Raman signals of AB via the chemical enhancement effect can be largely neglected. It is therefore reasonable to assume that the increased Raman scattering efficiency of AB is attributed to electromagnetic field modulation provided by the TiN support.

This is also supported by the conducted electromagnetic field calculations. In fact, calculated average EFs were comparable to the experimentally derived values from the SERS experiment and, more importantly, shared the same wavelength dependence. Specifically, the calculations revealed a weaker EF to be expected at the 430 nm peak with respect to the 700 nm peak in agreement with the experimentally observed lower REF at 413 nm excitation. The good match between the results from the near-field calculations and experimental SERS derived data further corroborates that the Raman signal amplification stems predominantly from light modulation effects by the TiN electrode.

Notably, the far-field simulations predicted the existence of two well-separated absorption peaks in agreement with the experimental UV-Vis spectrum of the nanostructured TiN. Considering the high prediction efficiency of the calculations also in the near-field case, it is reasonable to adapt the peak assignment derived from the theoretical investigation. In this respect, the lower wavelength peak at ca. 430 nm is proposed to represent a shifted SPP mode while the more intense higher wavelength peak at ca. 700 nm represents collective plasmon resonances arising from lattice resonances. This is partly in line with previous literature reports. In fact, while for most TiN nanostructures LPSRs have been found to result in one broad absorption peak, more recently, more ordered structures with high aspect ratios, specifically randomly oriented [11] and ordered tilted [28] TiN nanorods have also been shown to exhibit two absorption peaks. Here, the higher wavelength peaks were also proposed to originate from collective plasmonic coupling modes. The observation of such a collective plasmonic mode for the presented TiN nanostructure indicates a certain degree of order, such as local periodicity, to be present within the partially collapsed nanotube structure.

## 5. Conclusions

We presented the fabrication of plasmonic TiN electrodes with partially collapsed nanotubular structure. To assess the electromagnetic near-field enhancement of the electrodes, we established a facile experimental procedure featuring SERS spectroscopy and using a non-surface-attaching molecule as field reporter group. We showed that, in this case, the wavelength-dependent electromagnetic field enhancement of the TiN electrodes can be directly derived from the Raman signal enhancement of the probe at the respective wavelength. The SERS-based approach allows for rapidly obtaining realistic values for the near-field enhancement of nanostructured materials, also beyond TiN if the field reporter group is chosen properly. As such, the presented method will be particularly useful for rapid assessment of the capability nanostructured materials to operate as optical sensors that rely on light-enhancement and the investigation of such systems. Finally, we successfully employed rigorous coupled wave analysis for computing the optical responses of TiN. The experimental and computational results indicated that TiN nanostructures with high aspect ratios along with a certain degree of local periodicity are capable of hosting plasmonic lattice resonances that can significantly increase light amplification by the material and hence their operational capability as optical sensors.

## Data Availability

The data presented in this study are openly available in https://opara.zih.tu-dresden.de/xmlui/ (accessed on 20 December 2021), reference number 2035.

## Supplementary Materials

The following supporting information can be downloaded at https://www.mdpi.com/article/10.3390/s22020487/s1, Figure S1: Raman spectrum of the TiN electrode, Figure S2: SEM image of the TiO2 nanotube electrode and the TiN nanostructured electrode., Figure S3: Cyclic voltammogram of the AB loaded TiN electrode, Figure S4: 647 nm Raman spectra of AB in solution, Figure S5: Profile of the laser beam, Figure S6: Calculated field enhancement created by 4 TiN nanotubes in top view, Figure S7: Calculated field enhancement of 3 TiN nanotube in side view, Figure S8: Calculated relative absorbance and field enhancement factors as a function of TiN nanotube length, Figure S9: Calculated absorbance spectrum of a flat and a nanostructured TiN electrode.
