# Supplementary material for: Electromagnetic Field Enhancement of Nanostructured TiN Electrodes Probed with Surface-Enhanced Raman Spectroscopy
Source: Sensors (Basel). 2022 Jan 9;22(2):487. doi: 10.3390/s22020487 (PMC8781945; doi:10.3390/s22020487)
Supplement: Supplementary file 1 [file sensors-22-00487-s001.zip › sensors-1481439-supplementary.pdf]

Supporting Information

Electromagnetic Field Enhancement of Nanostructured TiN Electrodes Probed with Surface-Enhanced Raman Spectroscopy

Ibrahim Halil Öner [1,†], Christin David [2,†], Christine Joy Querebillo [1], Inez M. Weidinger [1,*] and Khoa Hoang Ly [1,*]

[1] Fakultät für Chemie und Lebensmittelchemie, Technische Universität Dresden, Andreas-Schubert-Bau, Zellescher Weg 19, 01069 Dresden, Germany; ibrahim.halil.oener@me.com (I.H.Ö.); christine_joy.querebillo@tu-dresden.de (C.J.Q.)

[2] Abbe Center of Photonics, Institute of Condensed Matter Theory and Optics, Friedrich-Schiller-Universität Jena, Max-Wien-Platz 1, 07743 Jena, Germany; christin.david@uni-jena.de

* Correspondence: inez.weidinger@tu-dresden.de (I.M.W.); hoang_khoa.ly@tu-dresden.de (K.H.L.)

† These authors contributed equally.

Table of content

Number	Section	Page
**1**	**TiN synthesis & characterisation**	
Figure S1	(A) Photo of the smooth and nanostructured TiN electrodes. (B) Raman spectrum of the rough TiN electrode.	3
Figure S2	SEM image of the nanotubular TiO_2 electrode (A) used as template for nitridation and subsequently obtained TiN electrode (B).	3
**2**	**Raman & surface enhance Raman spectroscopy of azidobenzene**	
Figure S3	Cyclic voltammograms of the TiN electrode before (trace a) and after (trace b) AB physisorption. Trace c is obtained after washing of the electrode with water.	4
Figure S4	Raman spectra of AB in tert-butylmethylether as a function of concentration. (B) n7 and n11 intensity as a function of AB concentration.	4
Figure S5	Approximated laser profiles for Raman/SERS solution (A) and surface (B) measurements.	5
**3**	**RCWA electromagnetic field calculations**	
Figure S6	Top view showing the distribution of localized field hot spots upon illumination at 413 nm for 6 different nanotube lengths *d*. Scale shows the field enhancement (EF) value to forth order $\|E\|^4/\|E_0\|^4$.	6
Figure S7	Side view of 3 units cells combined at illumination with 413 nm. The scale shows the field enhancement factor ($\boldsymbol{EF}$) value to forth order $\|E\|^4/\|E_0\|^4$.	7
Figure S8	(A) Calculated relative absorbance $A = 1 - R - T$ (A) and EME $\|\vec{E}\|^4/\|\vec{E}_0\|^4$ values (B) at fixed tube lengths *d* ranging from 160 to 300 nm. Dashed orange lines represent the average over the different nanotube lengths.	8
Figure S9	Spectral position of the plasmonic resonance of the flat TiN surface (black curve, SPP) compared to the nanostructured TiN electrode (*d* = 250 nm) exhibiting, in addition, a lattice resonance above 600 nm (green curve).	8

1. TiN Synthesis & Characterisation

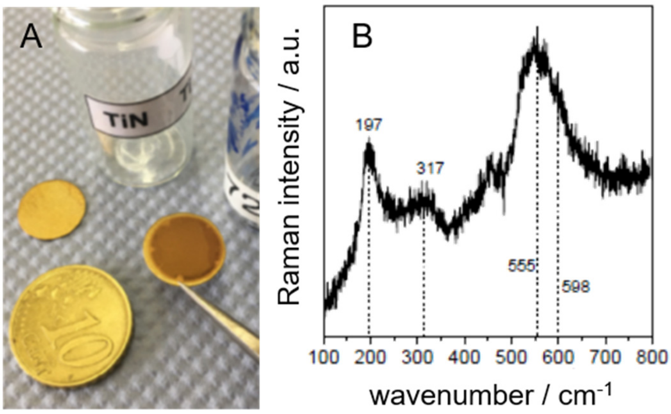

Figure S1. (**A**) Image showing the smooth (left) and nanostructured TiN electrode (right), respectively. (**B**) 514 nm Raman spectrum of the rough TiN electrode.

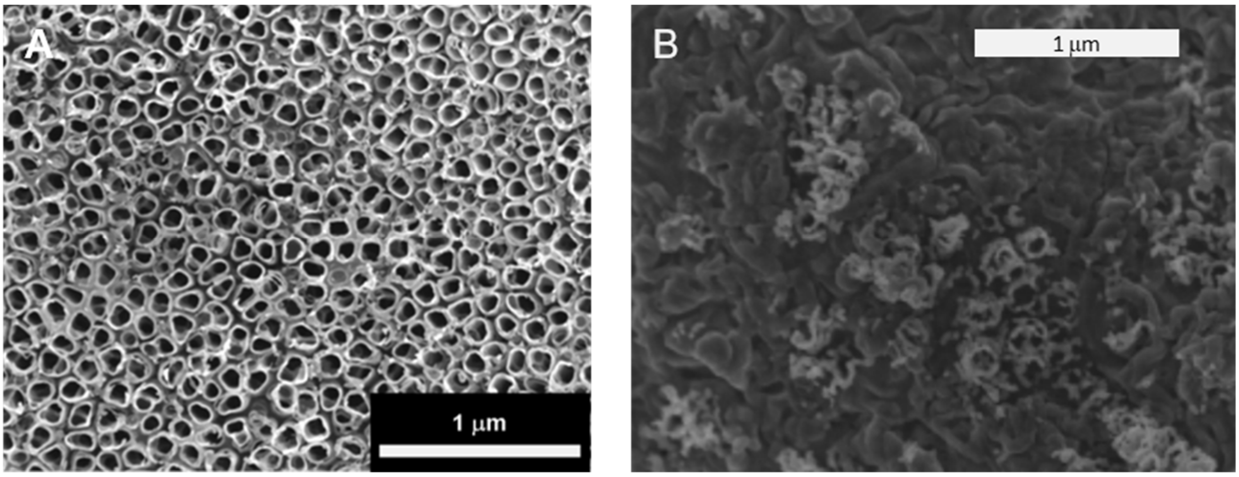

Figure S2. (**A**) SEM image of the nanotubular TiO_2 electrode used as template for nitridation. (**B**) SEM image of the subsequently obtained TiN electrode (B).

2. Raman & Surface-Enhanced Raman Spectroscopy of Azidobenzene

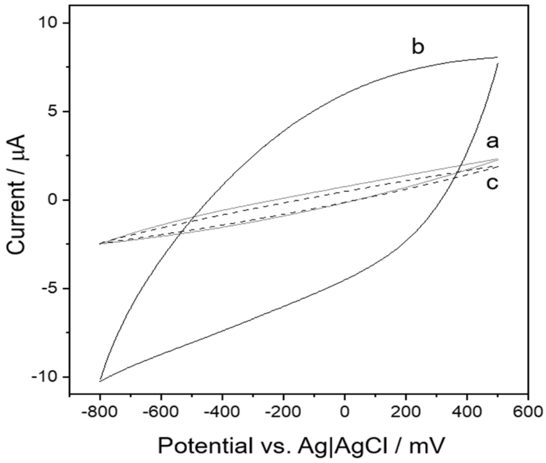

Figure S3. Cyclic voltammograms of the TiN electrode before (trace **a**) and after (trace **b**) reversible AB physisorption of azidobenzene. Trace **c** is obtained after washing of the electrode with water, indicating the reversibility of AB adsorption.

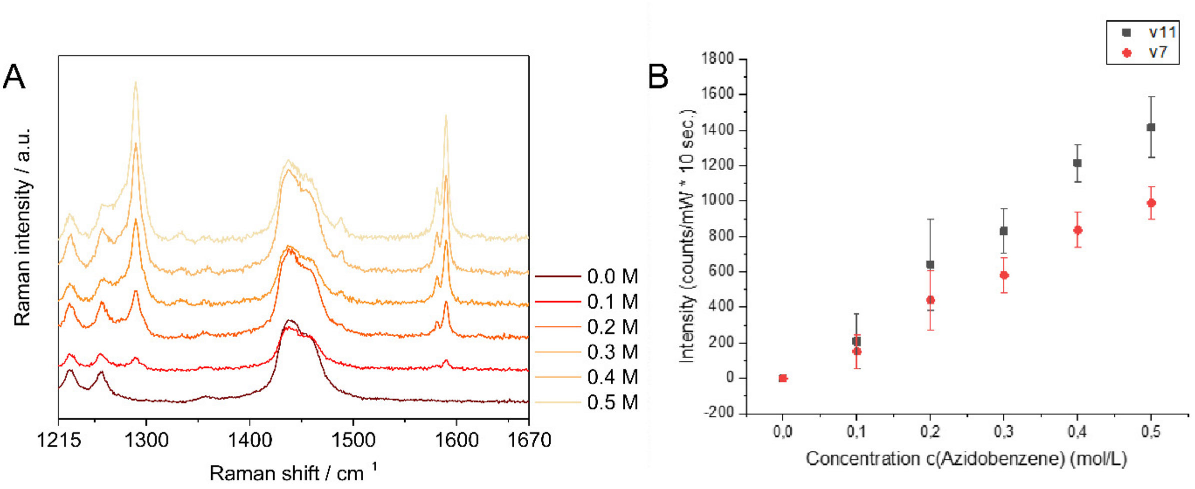

Figure S4. (A) 647 nm Raman spectra of azidobenzol in tert-butylmethylether. (B) Raman intensity of the ν_7 and ν_{11} band of azidobenzene as a function of concentration.

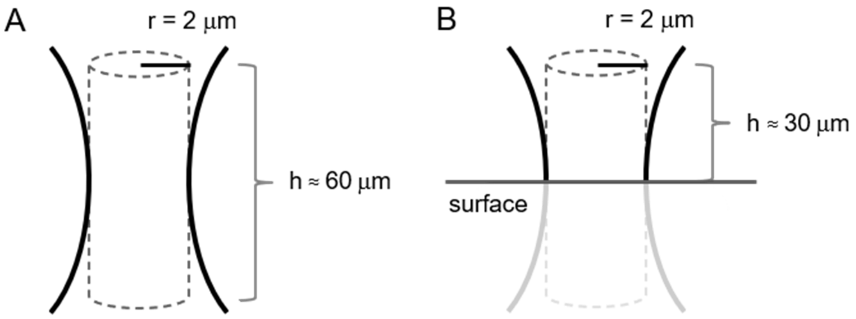

Figure S5. Approximated profile of the focussed laser beam for confocal Raman/SERS measurement in solution (A) and on the surface (B).

3. Electromagnetic Field Calculations Using RWCA

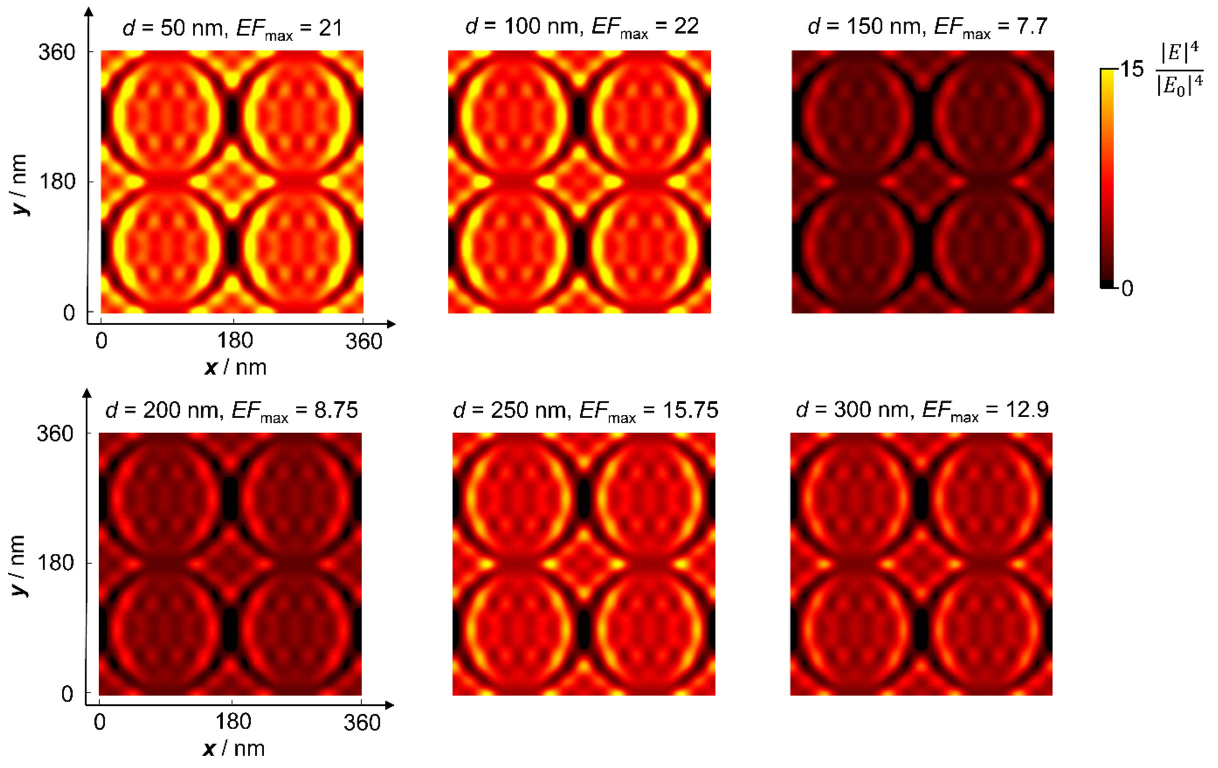

Figure S6. Top view over four unit cells showing the distribution of localized field hot spots upon illumination at 413 nm for six different nanotube lengths d. The scale shows the field enhancement (EF) value to forth order $|E|^4/|E_0|^4$.

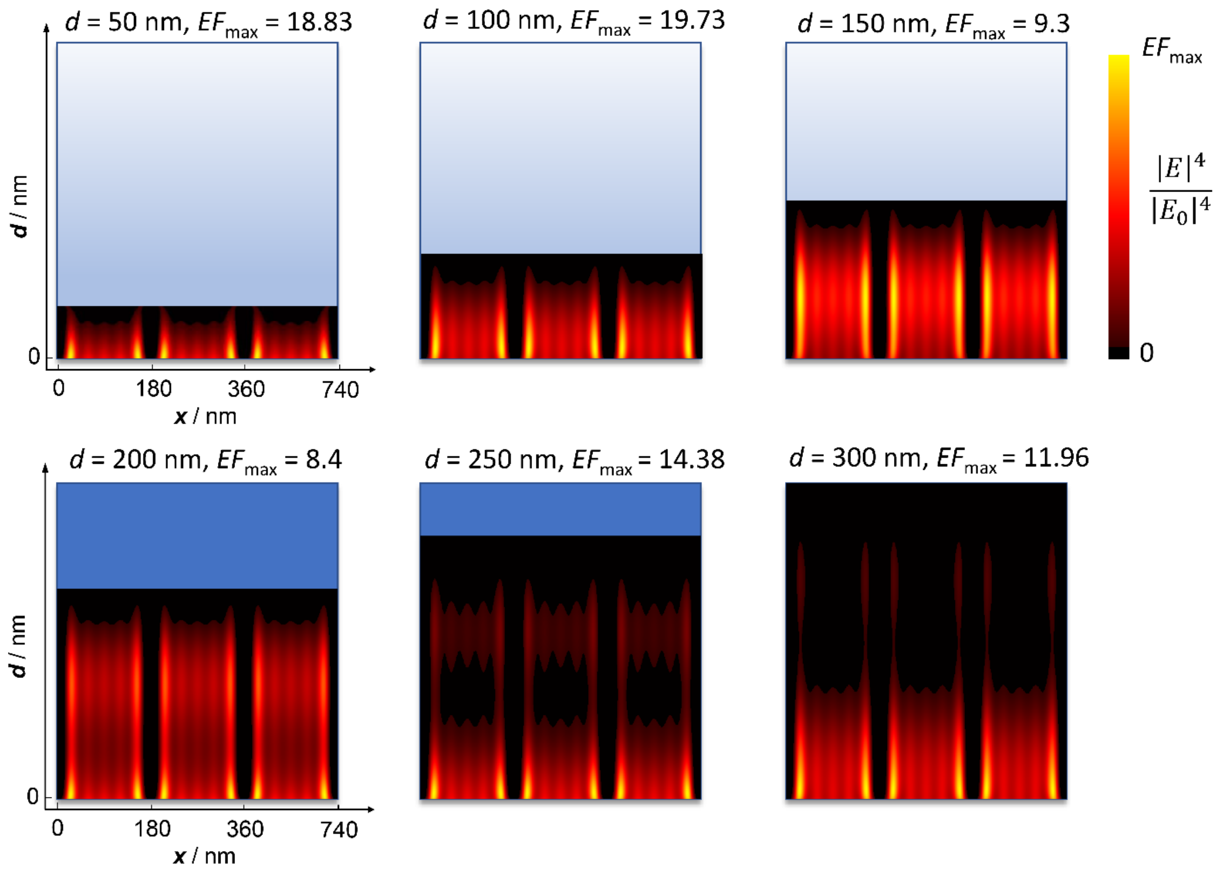

Figure S7. Side view of three units cells combined at illumination with 413 nm. The scale shows the field enhancement factor (*EF*) value to forth order $|E|^4/|E_0|^4$.

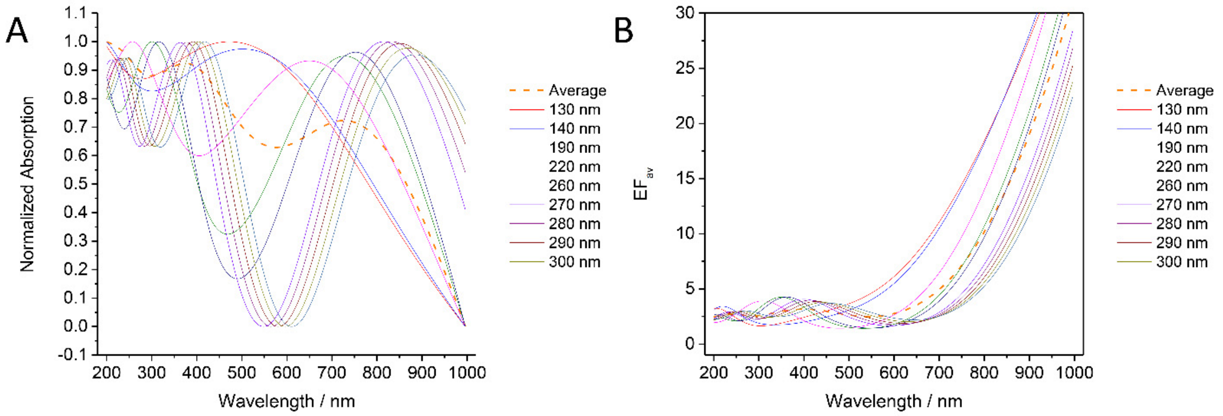

Figure S8. (A) Calculated relative absorbance $A = 1 - R - T$ (A) and EME $\frac{|\vec{E}|^4}{|\vec{E}_0|^4}$ values (B) at fixed tube lengths d ranging from 160 to 300 nm. The dashed orange lines represent the average over the different nanotube lengths.

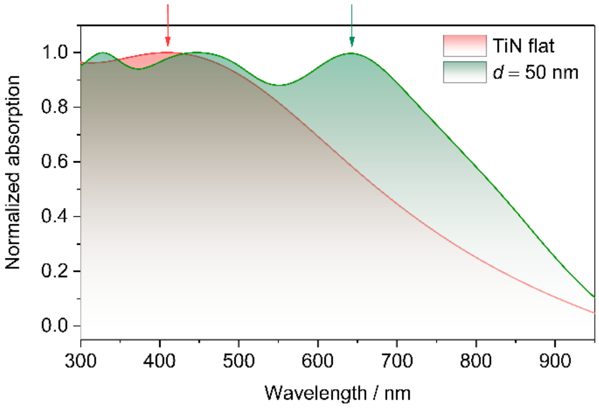

Figure S9. Spectral position of the plasmonic resonance of the flat TiN surface (red curve, surface plasmon polariton) compared to the nanostructured TiN electrode (d = 250 nm) exhibiting, in addition, a plasmonic lattice resonance peak above 600 nm (green curve).
